# Notes and News

**Published:** 1893-05-13

**Authors:** 


### Notes and News.

Collected and Communicated.

At the meeting of the London and Counties' Medical Protection Society, on Monday, 24th ult., Dr. Mead reported successful meetings and new branches formed in Cambridge, Bury St. Edmunds, and West London. Several examples of useful work by the Society were quoted, and Dr. Woods reported cases where Indian oculists had cut portions of the conjunctiva from the eyes of persons hopelessly blind from optic atrophy, leading them to suppose that they were removing a "skin," to which blindness was due, and obtaining considerable sums of money by promising a certain cure, which they have, of course, failed to effect. He stated that there were more than twelve of these oculists practising, and that they met together immediately after an inquest at Hackney on a child treated by one of their methods.

We are glad to learn that that excellent little institution, the Mary Wardell Home for Scarlet Fever Patients, at Stanmore, has, notwithstanding various vicissitudes, passed through a favourable year. The number of patients has been considerably larger than for several years, in spite of the closing of the Home for necessary cleansing and repairs. The income, too, shows a considerable increase, and from the source which testifies most to the appreciation of the Home, namely, from the patients themselves. The report justly points out that a season of epidemic such as was experienced last year should have called forth a far greater response from the public than was the case. Those immediately acquainted with the work of the institution help heartily. Dr. Little, who for years has attended the patients at the Home, has foregone the extra fees for constant attendance, required by the rules of the Home. Another friend has provided funds to assist patients to remain longer than they themselves could afford, and Mr. Zuppinger continues to supply the Home with flour. Many friends have sent welcome presents during the year. We should imagine that books and papers would be especially welcomed.

Amongst the list of wonderfully cheap production by way of Guides to London of all kinds, the "Handy Guide to May Meetings," published by E. May, Fleet Street, for one penny, should certainly hold a foremost place. It consists of 88 pages, in which are to be found information as to meetings, chariti s, trade information, literature, exhibitions, cab fares, and other matters besides, so that there are few who cannot gain some information from its pages, worth more to each individual than the humble penny asked.

The public meeting in aid of the fourth quinquennial appeal on behalf of the London Hospital took place on Thursday at the Mansion House, the Lord Mayor in the chair. A full report will appear in our next issue. The London, with its 780 beds, is the largest hospital in England, and depends for the most part on voluntary contributions for its support. Its expenditure reaches nearly £60,000, while its assured income is only £20,000 per year. It is built in the heart of Whitechapel, and surrounded by crowded districts, and the demands on its resources are extremely heavy. The fourth quinquennial appeal is now being made, and the Committee earnestly solicits support and help. To raise so large a sum as is required, it is natural that there must be some large donations and subscriptions, but it is earnestly hoped that those able and willing to give only a little will not be led, by the amount which has to be collected, to think that their contributions, however small, would not be appreciated. There are very many subscribers of a few shillings each year, and such help is most highly prized. All interested in the East-end are invited to visit the hospital, and any information will be gladly supplied by the House Governor.

Amongst the many interests of the World's Fair not one of the least will be the Donegal Village. The account of the village states that "It will be situated in the Midway Plaisance of the 'Street of Nations,' and it is now being erected by Mrs. Ernest Hart. The village will be by no means unpretentious, the conceded site covering an area of no less than 25,000 square feet. It stands between the Japanese village on the one hand and the great American glass works of Libby on the other. The entrance-gate of the village will be a replica of the St. Lawrence Gate at Drogheda, beyond which a hive of industry will be disclosed in the shape of a typical street of Irish cottages. Peasants from Donegal will occupy these, and will be engaged in one or other of the native industries which the Donegal Industrial Fund has done so much during the last ten years to foster and develop. They include lace-making, weaving, wood carving, embroidery work, the making of homespuns, &c. The ruins of Donegal Castle—one of the most beautiful in Ireland—will be thrown across the village, and they will include a faithful representation of the fine banqueting hall, 60 ft. by 30 ft., affording an excellent exhibition-room for embroideries and textile products. Through the archways of this ruin is entered a large courtyard, in the centre of which is a round tower 120 ft. in height, built after the style of the eighty or so still extant in Ireland, and the original purpose of which is still food for speculation. This tower has been presented to Mrs. Hart by Irish residents in America who sympathise with the work of the fund.

